# Introducing the YPIC challenge

**DOI:** 10.1016/j.euprot.2019.07.004

**Published:** 2019-08-14

**Authors:** Maarten Dhaenens

**Affiliations:** Ghent University, Ottergemsesteenweg 460, 9000, Gent, Belgium

There is nothing boring about science. Scientists are adventurers, shining light into the dark unknown that surrounds us. But what about a scientific challenge that is not directly aiming to elucidate new biology?

In 2016, the Young Proteomics Investigators Club (YPIC) did the thinking exercise: what if we could play science with no strings attached? For a European organization that wants to connect young scientists and help unfold their full potential, a scientific challenge in the form of a game is the ideal icebreaker.

**Figure:**
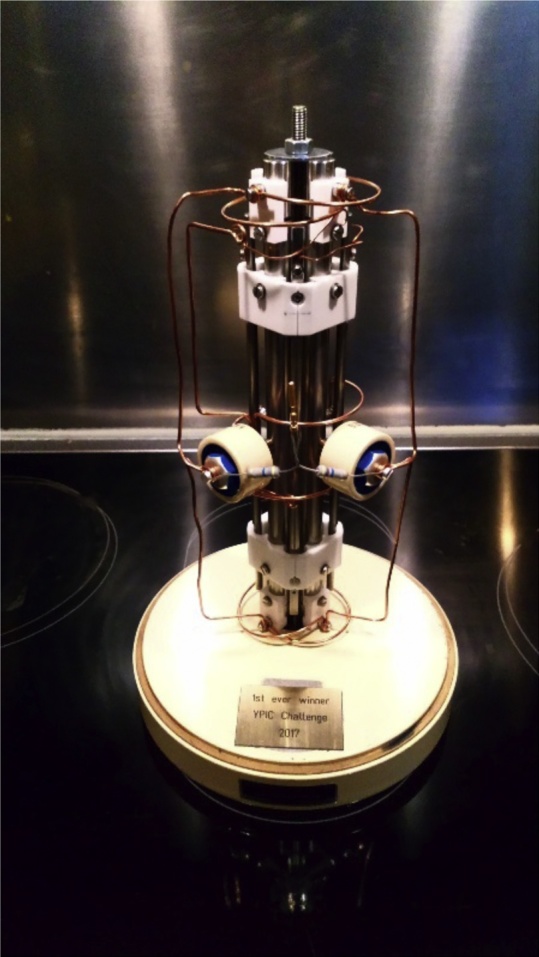

The one thing all the young scientists have in common is their fascination for proteins and peptides. To consolidate this solidarity, the **first edition of the YPIC challenge** incited the members to team up with complementary colleagues (mass spectrometrists, bioinformaticians, wet lab scientist…). When registered, a mixture of synthetic peptides made at ISAS by Ingo Feldmann (Prof. Dr. Albert Sickmann) was sent to them, which they had to analyze (no restrictions) in order to decipher the sentence formed by those peptides… It was not so much about finding all the words encoded in these peptides, as it was about finding the book where this quotation comes from. Just as in biology, you never elucidate every little detail, but you need to make conclusions about the underlying biological processes. Six out of the nineteen teams could crack the code and they presented their methodologies and results in a short manuscript. Some teams even found that one word (i.e. amino acid stretch) was missing in the peptide mixture! At HUPO 2017 in Dublin, the winning team was announced in a special session in the main hall. **Alexander Hogrebe and Rosa Jersie-Christensen** were awarded the very first YPIC Challenge cup. Their PI (Jesper Olsen) proudly accepted the quadrupole!

**Figure:**
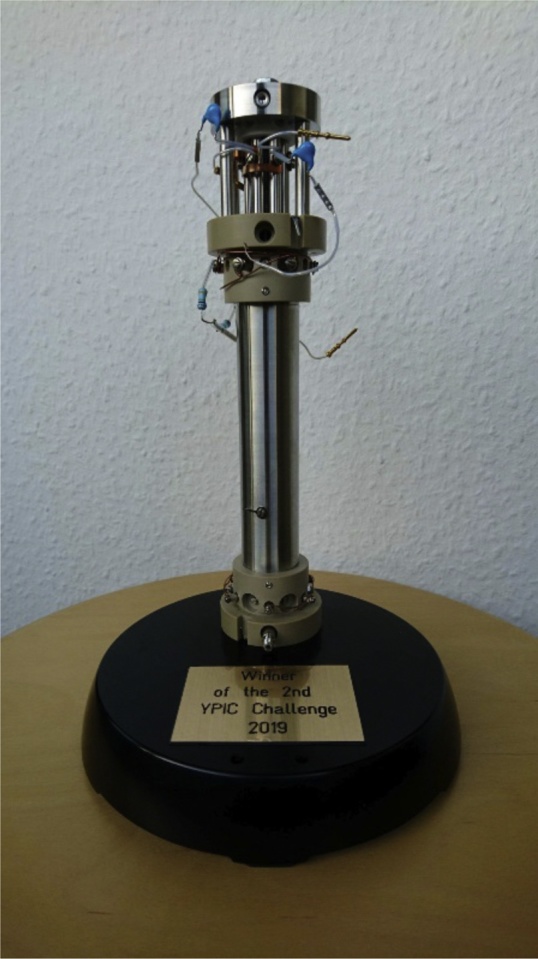

It goes without saying that YPIC had to take it up a notch for **the second challenge**. This time, they actually expressed the first-ever English sentence in E. coli, with the help of PolyQuant! You can read PolyQuant’s view on this adventure in their contribution in this Special Issues. At this point, the line between game, science and philosophy actually started blurring. Many things have been expressed in E. coli before, but an English sentence?! Chances are that English is in fact toxic to prokaryotes! Maybe certain grammatical constructions are bioactive or even cure cancer? Not to mention the international consequences when secret agencies start applying E. coli strains for encoding secret messages. It really becomes a philosophical question at this point: “Have you ever wondered what the most fundamental limitations in life are? Is there a structure to respect when it comes to what you can produce in a cell?” Well, two teams got exactly that question asked to them by E. coli! In the end, **Lili Liu and Matthias Mann** were awarded the second YPIC Challenge quadrupole cup at the Potsdam Proteomics forum (EuPA 2019).

**In this special issue of EuPA Open Proteomics, we bundle all these manuscripts**, as a tribute to the brave young scientists that worked until late at night to solve these mysteries. Two manuscripts are already published elsewhere: “The author identified by his method: EuPA YPIC challenge solved” by M.I. Indeykina D.A., Podgrudkov and A.S. Kononikhin (doi: 10.1016/j.euprot.2018.10.001) and “2018 YPIC Challenge: A case study in characterizing an unknown protein sample” by P. Lindsay, L. Andy and W. Bittremieux. You can read the latter work in the Journal of Proteome Research.

Enjoy reading their scientifically sound, yet hilarious output!

